# Effects of calcitriol (1, 25-dihydroxy-vitamin D3) on the inflammatory response induced by H9N2 influenza virus infection in human lung A549 epithelial cells and in mice

**DOI:** 10.1186/s12985-017-0683-y

**Published:** 2017-01-23

**Authors:** Boxiang Gui, Qin Chen, Chuanxia Hu, Caihui Zhu, Guimei He

**Affiliations:** School of Life Sciences, East China Normal University, Shanghai, 200062 People’s Republic of China

**Keywords:** Influenza, Calcitriol, Inflammation response

## Abstract

**Background:**

H9N2 influenza viruses circulate globally and are considered to have pandemic potential. The hyper-inflammatory response elicited by these viruses is thought to contribute to disease severity. Calcitriol plays an important role in modulating the immune response to viral infections. However, its unknown whether calcitriol can attenuate the inflammatory response elicited by H9N2 influenza virus infection.

**Methods:**

Human lung A549 epithelial cells were treated with calcitriol (100 nM) and then infected with an H9N2 influenza virus, or infected and then treated with calcitriol (30 nM). Culture supernatants were collected every 24 h post infection and the viral growth kinetics and inflammatory response were evaluated. Calcitriol (5 mg/kg) was administered daily by intraperitoneal injection to BABL/c mice for 15 days following H9N2 influenza virus infection. Mice were monitored for clinical signs of disease, lung pathology and inflammatory responses.

**Results:**

Calcitriol treatment prior to and post infection with H9N2 influenza significantly decreased expression of the influenza M gene, IL-6, and IFN-β in A549 cells, but did not affect virus replication. In vivo, we found that calcitriol treatment significantly downregulated pulmonary inflammation in mice 2 days post-infection, but increased the inflammatory response 4 to 6 days post-infection. In contrast, the antiviral cytokine IFN-β was significantly higher in calcitriol-treated mice than in the untreated infected mice at 2 days post-infection, but lower than in untreated infected mice on days 4 and 8 post-infection. The elevated levels of pro-inflammatory cytokines and the decreased levels of antiviral cytokine are consistent with the period of maximum body weight loss and the lung damage in calcitriol-treated mice.

**Conclusions:**

These results suggest that calcitriol treatment might have a negative impact on the innate immune response elicited by H9N2 infection in mice, especially at the later stage of influenza virus infection. This study will provide some novel insights into the use of calcitriol to modulate the inflammatory response elicited by influenza virus infection in humans.

## Background

Calcitriol, also known as 1, 25-dihydroxy-vitamin D3 (1, 25-(OH)2D3), is the biologically active form of vitamin D [1, 2]. The association between vitamin D and calcium homeostasis and bone metabolism is well established, but recently vitamin D has also been shown to regulate the immune system [1, 3], suggesting that understanding the pleotropic effects of vitamin D is important. The biological effects of 1,25-(OH)2D3 are mediated by the vitamin D receptor (VDR), which is expressed on many immune cells. Vitamin D affects cytokine production during the innate immune response, which also affects the subsequent adaptive immune responses [4].

Vitamin D deficiency is now recognized as a pandemic [5] and has been associated with an increased risk of respiratory infections. Vitamin D supplements reduce the risk of acute respiratory infection [6, 7]. Many observational studies have shown that vitamin D status is inversely associated with the prevalence of common colds [8–10]. Calcitriol treatment in human pulmonary epithelial cells (A549) before or after infection with an H1N1 influenza virus did not affect A549 cell viability, viral clearance or the anti-viral state following infection. However, expression of pro-inflammatory cytokines and chemokines was reduced at the genetic level [11], which may help alleviate the severity of the disease. In contrast, treatment with 1,25(OH)2D3 did not affect the expression pattern of human pro-inflammatory and anti-inflammatory responses elicited by viral pattern recognition receptor ligands at either physiologic or pharmacologic concentrations [12]. In addition, there are reports that vitamin D supplements do not increase the humoral immune to the influenza vaccine [13, 14]. Therefore, it remains unclear what effects vitamin D supplements have on the immune response to viral infection.

H9N2 subtype influenza viruses are the principal influenza strain reported in poultry and has circulated globally since their first detection in turkeys in 1966 [15]. They have also been detected in multiple other species [16]. In mammalian hosts, the H9N2 influenza viruses have been shown to provide internal genes to other influenza subtypes to create novel genotypes, such as H7N9 and H5N1 [17, 18]. Therefore, the H9N2 subtype influenza viruses play a significant role in the evolution of new influenza strains and are considered to have pandemic potential by the World Health Organization [19, 20]. H9N2 viruses were founded to elicit a markedly higher expression of inflammatory chemokines and cytokines than seasonal influenza viruses, which might contribute to the more severe disease caused by H9N2 viruses [20, 21]. Thus, immunomodulatory treatment strategies for influenza that suppress a hyper-inflammatory response may benefit influenza patients.

In this study, we hypothesized that calcitriol would reduce the inflammatory response elicited by H9N2 infection. To test this hypothesis, we treated human lung A549 epithelial cells and BABL/c with calcitriol and then characterized the inflammatory response to infection with an H9N2 influenza strain.

## Methods

### Virus

The H9N2 influenza virus used in this study was isolated from a wild duck in 2011 by our laboratory, and is similar to the 2009 human H9N2 isolates (HK/35820/2009 and HK/33982/2009). The strain is capable of inducing acute lung injury in BALB/c mice [22]. The complete genome sequences (JQ901621, JQ901632, JQ901643, JQ901654, JQ901665, JQ901676, JQ901687, and JQ901698) of the virus can be obtained from GenBank (National Center for Biotechnology Information, Bethesda, MD). To generate a stock, the virus was inoculated into the allantoic cavity of 10-day-old specific pathogen free (SPF) chicken embryos (Beijing Laboratory Animal Research Center, China) and incubated at 37 °C for 72 h. The allantoic fluid was then collected and stored at −80 °C until further use. The 50% tissue culture infection dose (TCID50) was determined in A549 cells as in our previous study [22].

### Reagents and cell lines

Calcitriol (Sigma-Aldrich, St. Louis, MO) was dissolved in ethanol to prepare a stock solution (1 mg/mL) and stored at −80 °C until further use. The stock solution was diluted to the desired concentration for experiments using culture medium or 0.9% sterile saline.

The human alveolar epithelial cell line A549 was purchased from the Cell Bank of Chinese Academy of Sciences, Shanghai, China. The cells were cultured in Dulbecco's Modified Eagle Medium (DMEM) (HyClone) containing 10% Fetal Bovine Serum (FBS) (Gibco) and 1% penicillin-streptomycin (Gibco). The A549 cells were grown at 37 °C in a 5% CO2 atmosphere.

### The effect of calcitriol on A549 cells

A549 cells were grown in T-25 tissue culture flasks (Corning) to approximately 75% confluence prior to use in experiments. The methods of measuring the effects of calcitriol pre- or post-treatment on the A549 cells were based on previous studies [11]. Briefly, calcitriol pre-treated A549 cells were incubated with calcitriol (100nM) for 16 h and then infected with H9N2 (1 mL volume, 1:200 dilution from stock, TCID50 = 106.7) for 1 h at 37 °C in a 5% CO2 atmosphere. After 1 h, the virus containing medium was removed and the cells were washed twice by PBS, then fresh DMEM containing 2 × pen-strep but no FBS was added and the cells were incubated at 37 °C in a 5% CO2 atmosphere for an additional 72 h. For cells that were treated with calcitriol after infection, virus infection was performed as described above and after the virus containing medium was removed, the infected cells were incubated with calcitriol (30nM) 72 h. The cell culture supernatants were harvested 24, 48, and 72 h post infection and the cell debris was removed by centrifugation. The TCID50 of the virus in the supernatant was determined in A549 cells to quantify the growth kinetics of the influenza virus in the absence and presence of calcitriol. Real-time PCR was performed to detect the expression levels of the influenza M gene, IL-6, and IFN-β.

### Mice

Specific-pathogen-free BALB/c female mice (aged 6–8 weeks) were purchased from Nanjing Pengsheng Biological Technology Co., Ltd. (China). Animals were maintained according to the National Institutes of Health (NIH) standards established in the Guidelines for the Care and Use of Experimental Animals, and all of the experimental protocols were reviewed and approved by the Animal Investigation Committee of East China Normal University. During the experiments, mice were provided ad libitum access to food and water in an environmentally controlled atmosphere with a 12-h light/dark cycle.

### Assessing the tolerability of calcitriol treatment in mice

To investigate whether there were any side effects of calcitriol treatment in mice, animals were divided randomly into a control or calcitriol treated group (*n* = 6 mice/group). The calcitriol-treated mice received intraperitoneal injections of calcitriol (5 mg/kg body weight) daily for 2 weeks [23, 24]. The control group received an intraperitoneal injection of an equivalent volume of sterile saline daily. Clinical signs (including body weight change, inactivity and mortality) and behavior were monitored daily for 2 weeks.

### Assessing the effects of calcitriol treatment on the course of influenza infection in mice

To study the effects of calcitriol supplementation on H9N2 virus-induced lung injury, BALB/c mice were randomly divided into an infected control group, calcitriol-treated infected group, and uninfected control group (*n* = 30 mice/group). Animals were lightly anaesthetized with diethyl ether, and mice in the infected control group and the calcitriol-treated infected group were inoculated intranasally with 100 μL of the H9N2 influenza virus A/mallard/Jiangxi/39/2011 allantoic fluid (1 × 106 50% egg infection dose), while the uninfected control group mice were inoculated intranasally (100 μL) with an equivalent dilution of noninfectious allantoic fluid. Mice in all groups were administered calcitriol or saline starting the next day, and their general behavior and clinical signs were monitored daily. Five mice from each group were euthanized on 2, 4, 6, 8 and 15 days post-infection. The whole l ungs were harvested and stored at −80 °C for histopathology observation, inflammatory cytokine and viral gene analysis. Mice were euthanized using CO2 when their weight loss was ≥25% of their initial body weight. All efforts were made to minimize animal suffering.

### Histopathology measurement

At the time points indicated, half lung lobes from each mouse were fixed in buffered 10% formalin for 7 days, then embedded in paraffin, sectioned (5 μm slices), and stained with hematoxylin-eosin. All samples were randomly numbered and examined using light microscopy by a blinded veterinary pathologist [25]. The total lung histopathological scores for each section were calculated by adding the separate scores. The change in the histopathological scores over time was expressed as the mean ± SEM at each time point.

### Real-time quantitative PCR analysis

The expression levels of IL-2, IL-4, IL-6, TNF-α, IFN-β,VDR and the H9N2 M gene were assessed by relative quantitative real-time PCR (qPCR) performed using the Applied Biosystems 7300 system (Life Technologies, Carlsbad, CA) [26]. Total RNA was extracted from A549 cells and lung samples using the RNeasy Mini Kit (QIAGEN) per the manufacturer’s instructions. Random primers (Promega, Madison, WI) and Superscript III (Life Technologies) were used to synthesize first-strand complementary DNA from equivalent amounts of RNA. The qPCR primers were designed using Primer Express software (Life Technologies). The housekeeping gene β-actin was used as an internal control. The amplification efficiencies of the target genes and the β-actin gene were determined by serially diluting the cDNA and performing qPCR [27]. The amplification efficiencies were close to 100% (data not shown). qPCR was performed using the SYBR Green PCR master mix (Takara, Dalian, China) under the following reaction conditions: 95 °C for 30 s and 40 cycles of 95 °C for 5 s, 60 °C for 31 s, and a dissociation step. Each sample was amplified in quadruplicate. All data were analyzed using he Sequence Detector Systems software (Life Technologies). Relative quantification (RQ) was carried out using the 2-ΔΔCt method in which the average threshold cycle (ΔCt) values of the control group were used for calibration [28]. The sequences of primers used in this study are available upon request.

### Statistical analysis

All data were analyzed using the Statistical Package for Social Science version 17.0 (SPSS, Inc., Chicago, IL). Results are expressed as the mean ± SEM. The one-way analysis of variance (ANOVA) followed by a post-hoc Tukey test or unpaired two-tailed *t*-test was used to evaluate the statistical significance of differences between two groups. *P*-values less than 0.05 were considered statistically significant.

## Results

### The effects of calcitriol treatment in A549 cells

**Fig. 1:**
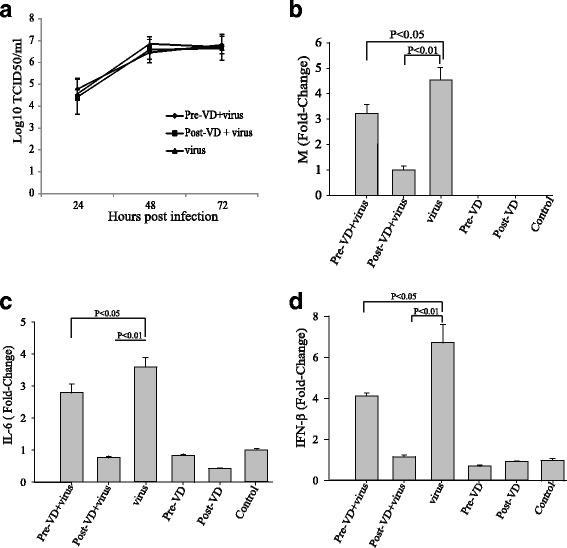
The effects of calcitriol on A549 cells. **a** Effect of calcitriol treatment on virus growth kinetic in A549 cells. Cell culture supernatants were harvested every 24 h from infected cells and the viral load was titrated using the TCID50 assay. Real-time PCR was used to measure the effect of calcitriol treatment on the mRNA expression levels of the viral M gene (**b**), IL-6 (**c**), and IFN-β (**d**) in A549 cells. β-actin was used as an internal control. The data are expressed as mean ± SEM of triplicate samples

We hypothesized that the immunomodulatory effects of calcitriol would attenuate the effects of H9N2 influenza in A549 cells. To establish that any observed attenuation was not the result of deficient viral growth, we first tested whether calcitriol treatment affected the growth kinetics of H9N2 influenza in A549 cells. The culture supernatants from infected cells were harvested every 24 h and the viral load was determined by TCID50 assay. The growth kinetics of H9N2 influenza in cells pre-treated with calcitriol or treated with calcitriol post infection were similar to that of infected cells in the absence of calcitriol (Fig. 1a). In all cases, the viral titer peaked at 48 h post infection and then plateaued. These results indicate that the replication competence of H9N2 influenza is not influenced by the calcitriol treatment.

Based on the viral growth kinetics, we selected the 48h post infection time point to assess the relative mRNA expression levels of the viral M gene, IL-6 and IFN-β by real-time PCR. Significant increases in the expression levels of the M gene (Fig. 1b), IL-6 (Fig. 1c) and IFN-β (Fig. 1d) were observed in A549 cells infected with H9N2 influenza compared with uninfected control cells, indicating that hyperinflammatory responses were elicited by H9N2 influenza virus infection. Treatment with calcitriol pre-infection and post-infection significantly reduced the expression of the M gene (Fig. 1b). In the uninfected cells, regardless of calcitriol treatment, no M gene expression was detected. Similar changes were observed with regard to expression levels of IL-6 and IFN-β in H9N2 infected A549 cells treated with calcitriol pre- or post- infection (Fig. 1c and d). Taken together, these results suggested that calcitriol might reduce the immune response to H9N2 influenza infection in A549 cells.

### Assessing the effects of calcitriol treatment in mice

**Fig. 2:**
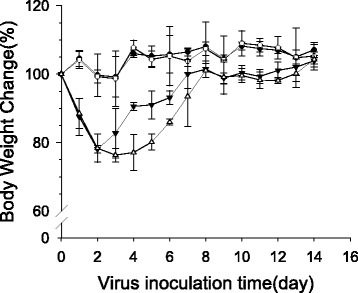
Body weight change in mice. White circles indicate mice inoculated with noninfectious allantoic fluid and treated with sterile saline from days 1 to 15 post-infection; Black circles indicate mice inoculated with noninfectious allantoic fluid and injected with calcitriol (5 mg/kg) intraperitoneally; White triangles indicate mice infected with the H9N2 virus and treated with calcitriol (5 mg/kg) from days 1 to 15 post-infection; Black triangles indicate mice infected with the H9N2 virus and treated with sterile saline from days 1 to 15 post-infection. Data presented are the mean ± SEM for 6 mice per group

Given that in vitro calcitriol appeared to dampen the inflammatory response to H9N2 infection in A549 cells, we then hypothesized that calcitriol treatment might attenuate the clinical symptoms of H9N2 infection in mice. To determine whether calcitriol administration was well tolerated in mice the clinical signs and body weight of uninfected mice treated with calcitriol were monitored daily. No changes were observed in either the body weight (Fig. 2) or clinical signs (data not shown) when comparing calcitriol-treated and untreated mice. Calcitriol administration was also not associated with morbidity. Together these results indicated that the 5 mg/kg body weight calcitriol dose chosen for this study was well tolerated.

We then compared the effects of H9N2 infection in mice treated with calcitriol to untreated mice. We found that body weight decreased in infected mice on days 1–2 after H9N2 virus infection irrespective of calcitriol treatment. Furthermore, on days 3–7 post-infection, infected mice also exhibited severe clinical signs, including marked inactivity, emaciation, ruffled fur, lack of appetite, and signs of labored breathing and respiratory distress (data not shown). The body weight loss was greater in the calcitriol-treated infected group than in the infected control group, but the difference was not significant (Fig. 2). The uninfected mice showed no clinical signs of infection. These findings indicated that daily treatment with calcitriol did not alleviate the clinic symptoms of H9N2 virus in infected mice.

### Lung histopathology and M gene expression in mice treated with calcitriol

**Fig. 3:**
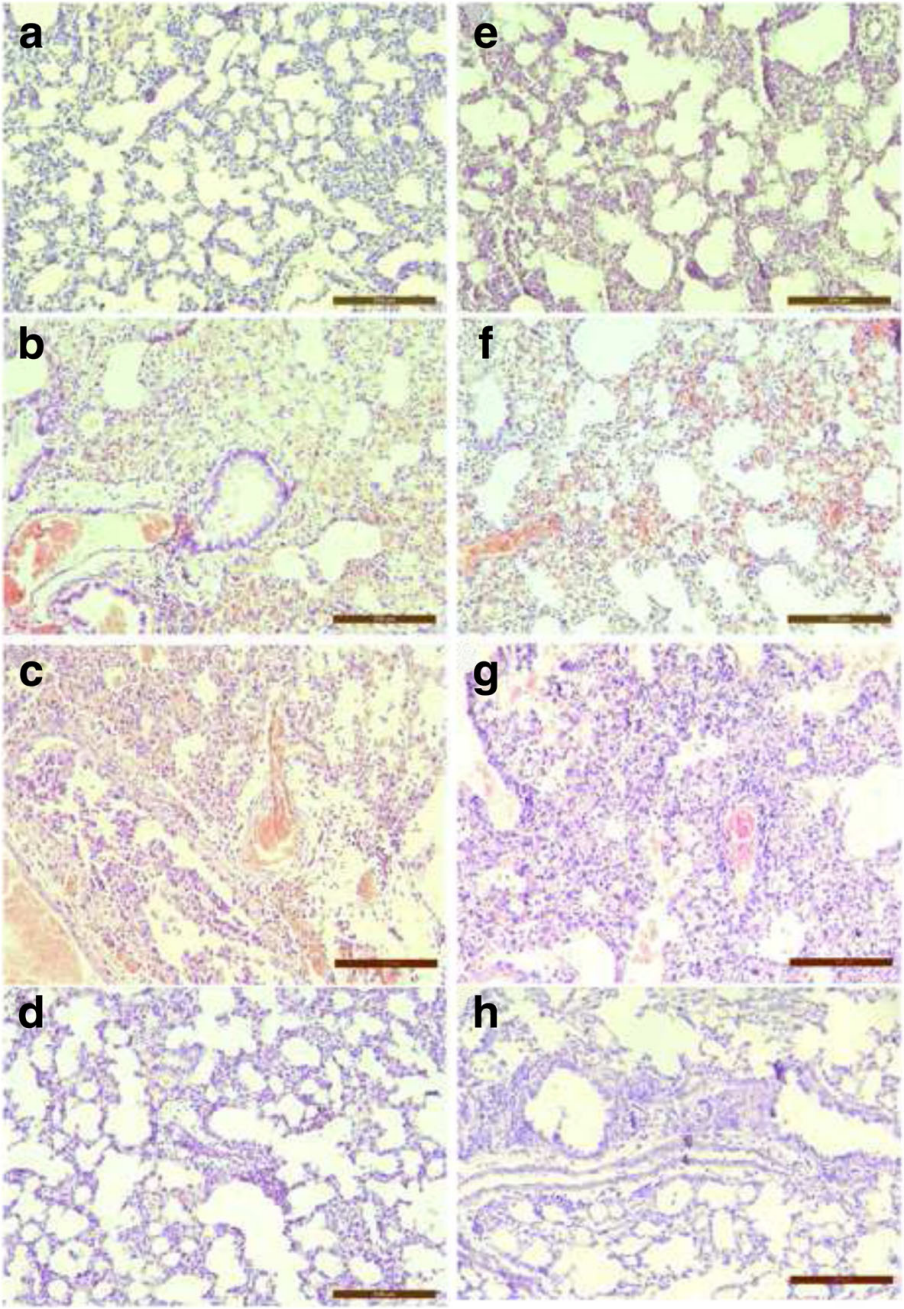
Changes in lung histopathology of calcitriol treated mice. Lung samples were harvested from calicitriol (5 mg/kg) treated H9N2 infected mice on days 2 (A), 4 (B), 6 (C), and 8 (D) post infection. Lung tissues were harvested from untreated H9N2 infected mice on days 2 (E), 4 (F), 6 (G), and 8 (H) post-infection. Original magnification 100 ×

To understand whether calcitriol treatment impacted lung pathology associated with H9N2 infection, lung samples were collected on days 2, 4, 6, and 8 post-infection and examined by light microscopy. The histopathology scores for mice infected with H9N2 influenza are provided in Table 1. Representative histology images are shown in Fig. 3. Regardless of calcitriol treatment, infected mice exhibited similar pathological patterns, including collapse of the alveolar space, infiltration of inflammatory cells, interstitial and alveolar edema, and hemorrhage. The change in the histopathology score for calcitriol-treated infected mice was less than the untreated infected mice 2 days post-infection, but greater on days 4 and 6 post-infection (Table 1 and Fig. 3). At later time points the infected mice had significant margination and infiltratory cells with profuse hemorrhage and edema. The results suggested that rather than attenuating H9N2 pathology, calcitriol treatment might exacerbate lung damage during the later stages of influenza virus infection.

**Table 1:** Histological examination of the lung tissue

Parameter	Days post-infection			
	2	4	6	8
Hemorrhage				
Infection^a^	0.87 ± 0.35	2.27 ± 0.45	2.12 ± 0.75	0.42 ± 0.5
Infection + Calcitriol^b^	0.78 ± 0.44	2.53 ± 0.51*	3.05 ± 0.58**	0.96 ± 0.81**
Alveolar and interstitial edema				
Infection^a^	1 ± 0.53	1.82 ± 0.59	2.08 ± 0.86	0.96 ± 0.53
Infection + Calcitriol^b^	1.11 ± 0.78	3.03 ± 0.64**	2.45 ± 0.6*	0.93 ± 0.38
Margination and infiltration				
Infection^a^	1.62 ± 0.52	2.77 ± 0.43	2.59 ± 0.67	1.73 ± 0.45
Infection + Calcitriol^b^	1.44 ± 0.53	3.25 ± 0.64*	3.5 ± 0.51**	1.52 ± 0.51
Alveolar collapse				
Infection^a^	1.75 ± 0.46	2.68 ± 0.48	2.96 ± 0.76	1.69 ± 0.47
Infection + Calcitriol^b^	1.22 ± 0.44	3.25 ± 0.52*	3.18 ± 0.5	1.37 ± 0.49
Total lung injury score				
Infection^a^	5.25 ± 1.16	9.54 ± 1.22	9.75 ± 2.38	4.81 ± 1.52
Infection + Calcitriol^b^	4.55 ± 1.67	12.1 ± 1.54**	12.2 ± 1.18**	4.78 ± 1.25

Data are presented as the mean ± SEM, unless otherwise stated. ^a^: Mice infected with the H9N2 virus and treated with sterile saline from days 1 to 15 post-infection; ^b^: Mice infected with the H9N2 virus and treated with calcitriol (5 mg/kg body weight) from days 1 to 15 post-infection; *P <0.05; **P <0.01

**Fig. 4:**
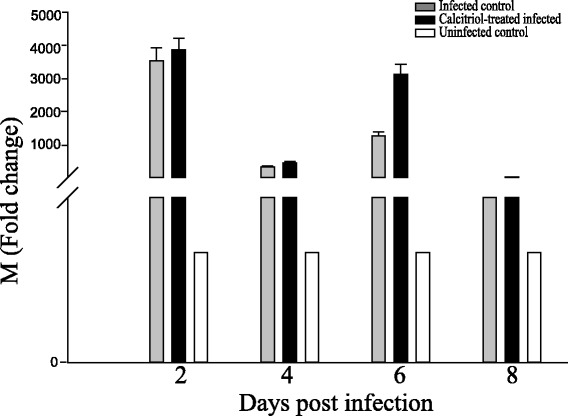
Expression of the H9N2 M gene in the lungs of infected mice after treatment with calcitriol. Mice were inoculated intranasally with 100 μL of allantoic fluid containing influenza A/mallard/Jiangxi/39/2011 (H9N2; 1 × 106 50% egg infection dose). Lung tissues were harvested on the indicated days post-infection. The relative quantification (RQ) values for the H9N2 M gene, are expressed as fold-change. RQ values were obtained using the 2-ΔΔCt method normalizing to the RNA expression levels of the β-actin gene. The average threshold cycle (ΔCt) values of the untreated infected group were used for calibration

The expression of the H9N2 M gene was measured by real-time quantitative PCR (Fig. 4) mRNA for the M gene was detectable from 2 to 8 days post-infection in all of the infected mice. However, expression levels were slightly higher in the calcitriol treated mice compared to the untreated infected mice from 2 to 6 days post-infection. Notably, expression levels in the calcitriol treated mice were twice that in the untreated infected mice 6 days post-infection. There were no differences between the two groups by 8 days post-infection. Consistent with the histology findings, these results suggested that calcitriol treatment might increase the expression levels of influenza M gene in the lungs.

### Expression of pro-inflammatory and antiviral cytokines in the lungs of calcitriol treated mice

**Fig. 5:**
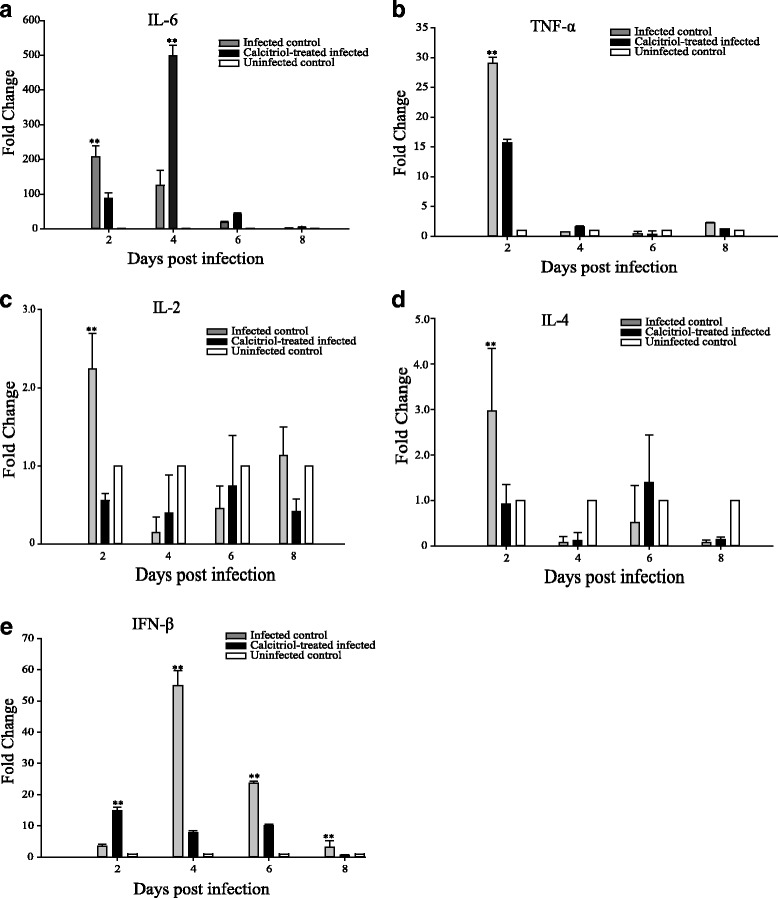
Expression of pro-inflammatory and antiviral cytokines in calcitriol treated mice. Expression levels of Interleukin (IL)-6 (A), tumor necrosis factor (TNF)-α (B), IL-2 (C), IL-4 (D) and IFN-β (E) were measured by real-time PCR in the lungs of mice treated with calcitriol. The relative quantification (RQ) values for these genes in calcitriol-treated infected mice were calculated, and the average threshold cycle (ΔCt) values of the infected control group were used for calibration

To investigate the effects of calcitriol treatment on the expression of pro-inflammatory cytokines (IL-2, IL-4, IL-6 and TNF-α) and antiviral cytokines (IFN-β) following H9N2 influenza virus infection, lung samples were harvested at the times indicated and the relative mRNA expression levels were calculated using the 2^-ΔΔCt^ method. Compared to uninfected mice, the expression levels of IL-2, IL-4, IL-6 and TNF-α in H9N2 influenza virus infected control mice were similar, which was elevated significantly on day 2 post-infection and reached a peak on this day and then began to decline on day 8 (Fig. 5a–d). Interestingly we found that the levels of TNF-α, IL-2 and IL-4 returned to baseline or were decreased compared to the uninfected control group by 4 to 6 days post-infection. The expression levels of IL-2, IL-4, IL-6 and TNF-α in calcitriol-treated infected mice were significantly lower than the untreated infected mice on 2 days post-infection (*p* <0.01). However, the expression levels were higher than the untreated infected mice on 4 and 6 days post-infection. In particular, the levels of IL-6 were upregulated up to 5.6 fold in calcitriol-treated infected mice at 4 days post-infection.

In the absence of calcitriol treatment, the expression levels of IFN-β in infected mice increased at 2 days post-infection, peaked at 4 days post-infection, and then began to decline. In contrast, to the proinflammatory cytokines, IFN-β expression levels were significantly higher in the calcitriol treated infected mice than in the untreated infected mice on days 2 post-infection, but significantly lower at 4 and 8 days post-infection (*p* <0.01; Fig. 5e).

### Calcitriol treatment upregulated expression of VDR in the lung

**Fig. 6:**
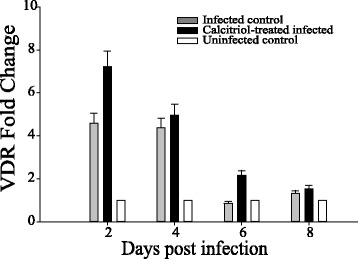
VDR expression in the lungs after calcitriol treatment. The relative quantification (RQ) values for vitamin D receptor (VDR) mRNA expression were calculated using the 2-ΔΔCt method normalized to the β-actin gene. The average threshold cycle (ΔCt) values of the infected control group were used for calibration

The biological effects of calcitriol are mediated by the vitamin D receptor (VDR). As shown in Fig. 6, the expression of VDR mRNA was greater in mice treated with calcitriol than in untreated mice, the greatest difference being approximately 2.5-fold on 6 days post-infection.

## Discussion

H9N2 viruses are known to elicit a hyperinflammatory reponse that includes cytokines such as IL-6 and TNF-a, that may play an important role in contributing to pathogenicity [20, 21, 29]. In A549 cells, we found that IL-6 and IFN-β were significantly upregulated response to H9N2 influenza virus infection. This is consistent with findings from avian H9N2 infection in A549 cells and TBE cells (tracheobronchial epithelial) eliciting a pro-inflammatory cytokine response including production of IL-6, IL-1β, and IFN-β [30, 31]. In vivo, we found that that the expression levels of IL-2, IL-4, IL-6 and TNF-a in the lungs of H9N2 infected mice peaked on 2 days post-infection and then declined on days 4 and 6 post-infection. Interestingly, the expression levels of IFN-β did not peak until 4 days post-infection and then declined. The increased cytokine levels were associated with the period of most dramatic body weight loss and the expression of the influenza M gene in the lungs. Therefore, the cytokine dysregulation in response to H9N2 influenza virus infection might contribute to the increased severity of the disease, especially in the early stages of the infection.

Vitamin D plays important roles in modulating the innate immune response to viral infection and can suppress the inflammatory response [32, 33]. We hypothesized that calcitriol would attenuate the inflammatory response induced by H9N2 influenza virus infection. In A549 cells we found that treatment with calcitriol prior to and post infection with H9N2 influenza significantly decreased the mRNA expression levels of IL-6, IFN-β and the viral M gene compared to infected cells not treated with calcitriol. Treatment did not affect H9N2 replication in A549 cells. This suggested that calcitriol treatment might downregulate the immune response to the H9N2 avian influenza virus infection in vitro assays.

To determine the effects of calcitriol treatment on the clinical course of H9N2 infection we used a mouse model and characterized the inflammatory response to infection. Calcitriol treatment did not attenuate the clinic symptoms of H9N2 infected mice, and in fact body weight loss was slight greater in the calcitriol-treated infected group than in the untreated infected group. While calcitriol treatment slightly attenuated the severity of the lung lesions in infected mice during early infection, it significantly aggravated lung damage in the later stage of influenza virus infection. Calcitriol treatment also increased the mRNA levels of the influenza M protein from 2 to 6 days post-infection. The increase in influenza M gene expression in calcitriol-treated mice was consistent with the increased disease severity. The M protein is not only directly related to virus replication, it also can lead to the activation of inflammasomes [34], and therefore we quantified the inflammatory response.

We characterized the inflammatory response by measuring the mRNA expression levels of pro-inflammatory and antiviral cytokines. Calcitriol treatment reduced the expression of the pro-inflammatory cytokine IL-6 at the earliest stages of infection, but increased its expression in the later stages of infection. In contrast, the antiviral cytokine IFN-β was significantly higher in calcitriol-treated mice than in the untreated infected mice at 2 days post-infection, but lower in the calcitriol treated mice on days 4 and 8 post-infection. The increase in pro-inflammatory IL-6 and the decrease in antiviral IFN-β are consistent with the changes the lung lesions in calcitriol-treated mice. Taken together these results suggest that calcitriol treatment might hamper the innate immune response to H9N2 influenza virus infection in mice, especially at the later stage of influenza virus infection. The changed we observed in the levels of IL-2 and IL-4 were similar to previous reports demonstrating that IL-2 and IL-4 were downregulated in chicken macrophages infected with an H9N2 avian influenza virus, This study suggested that cytokine downregulation might have an overall negative impact on the development of adaptive immunity in chickens [30]. While the expression levels of IL-2 and IL-4 in calcitriol treated mice were a slightly higher following infection than in untreated mice at days 4 to 6 post-infection, there were no marked differences between the three groups. Therefore, it is still unclear whether IL-2 and IL-4 play a negative role in the development of the adaptive immune response in this model.

Vitamin D binds to VDR, a steroid/thyroid hormone nuclear receptor, which has been identified on many immune cells, suggesting vitamin D may have a regulatory role in the immune system. To investigate whether the effects of calcitriol in mice may have been due to increased expression of VDR in the lungs we determined the level of VDR expression by PCR. Expression of VDR in the lungs of the calcitriol-treated infected mice was higher than in the untreated infected mice at the times indicated. Upregulation of VDR in response to vitamin D has also been observed in vivo [35]. Calcitriol treatment resulting in elevated VDR levels can be explained by a similar rise observed in the proinflammatory cytokines that triggers the hyper-responsiveness induced influenza virus infection. Therefore, VDR elevation might be involved in the inflammatory processes in vivo.

In summary, calcitriol has been shown to have a positive effect on the inflammatory response in vitro. However, daily treatment with calcitriol following H9N2 influenza virus infection did not attenuate the clinical severity of the disease, and in fact slightly increased the inflammatory response and the severity of lung injury during the later stage of infection. The reason for the discrepancy in the in vivo and in vitro studies is unclear. It may be that the differences in the responses are attributable to the complex role of vitamin D in vivo, which may affect one component of the immune system response but not other components. This complexity might make the net effect of calcitriol on immune function and clinical illness difficult to characterize and may suggest differences in immune regulatory mechanisms depending on the in vitro and in vivo systems used. In developed countries, dietary supplements containing vitamin D are widely used, and vitamin D supplementation is now common [36]. We know that the pathophysiology of avian influenza virus infection in mice is significantly different from that in humans. We hope these results will provide novel insights for additional studies into the anti-inflammatory role of calcitriol during influenza virus infection in humans.

## Conclusions

Herein, we hope this study will provide some insights into calcotriol treatment in modulating the inflammatory response induced by influenza virus infection in humans.
